# Periodontal Pathogens Correlate with Rheumatoid Arthritis Disease Parameters: A Systematic Review Based on Clinical Studies

**DOI:** 10.3390/dj13050214

**Published:** 2025-05-15

**Authors:** Luki Astuti, Sri Lelyati Chaidar Masulili, Indrayadi Gunardi, Benso Sulijaya, Yuniarti Soeroso

**Affiliations:** 1 Doctoral Program, Faculty of Dentistry, Universitas Indonesia, Jakarta 10430, Indonesia; luki.a@trisakti.ac.id; 2 Department of Periodontology, Faculty of Dentistry, Universitas Trisakti, Jakarta 11440, Indonesia; 3 Department of Periodontology, Faculty of Dentistry, Universitas Indonesia, Jakarta 10430, Indonesia; srilelyati@yahoo.com (S.L.C.M.); benso.sulijaya87@ac.ui.id (B.S.); 4 Department of Oral Medicine, Faculty of Dentistry, Universitas Trisakti, Jakarta 11440, Indonesia; indrayadi@trisakti.ac.id

**Keywords:** rheumatoid arthritis, periodontitis, microorganisms, disease parameters, autoantibodies

## Abstract

**Background**: Numerous studies have found higher levels of autoantibodies including anti citrullinated protein antibodies (ACPAs), anti-cyclic citrullinated peptides (aCCP), or rheumatoid factor (RF) against periodontal microorganisms in rheumatoid arthritis (RA). Objective: To evaluate the correlation between periodontal bacteria and RA disease parameters. **Methods**: We utilized PubMed, Scopus, ScienceDirect, and manual search databases up until March 2024 using PRISMA 2020 guidelines. The data were obtained from microbiological assays by RT-PCR/qPCR, sequencing, and serological testing of disease parameters (ACPA, aCCP, and RF) utilizing ELISA method. **Results**: A total of 1514 documents were discovered based on the inclusion criteria. *Porphyromonas gingivalis*, *Aggregatibacter actinomycetemcomitans*, and *Prevotella_9* were associated with elevated levels of ACPA/aCCP and RF in RA with periodontitis. A positive correlation was found between *Peptococcus simiae*, *Aminipila butyrica*, *Leptotrichia* spp., *Leptotrichia wadei*, and *Neisseria bacilliformis* with ACPA, and *Treponema* sp. canine oral taxon 087 with RF. **Conclusions**: This study found that several oral microorganisms correlate with elevated ACPA/aCCP and RF in RA with periodontitis. Future studies of the oral microbiome and the molecular mechanisms are anticipated to discover new therapies and diagnostic methods for periodontitis and RA.

## 1. Introduction

Rheumatoid arthritis (RA) and periodontitis (PD) are chronic inflammatory conditions that have been the subject of extensive research due to their potential interrelationship. The association between RA and PD has been a topic of interest, with studies suggesting a significant link between the two conditions. Both conditions share common pathophysiological mechanisms, and the potential influence of oral microbiome. RA, a chronic inflammatory disease, is caused by a combination of hereditary factors and environmental risks. One regularly recognized risk factor for developing RA is periodontitis [1,2,3,4,5,6]. Multiple studies have demonstrated that individuals with RA have elevated levels of autoantibodies against periodontal pathogens compared to control subjects [7]. Research by Smit et al. [8] and Konig et al. [9] found higher antibody titers against pathogen such as *Porphyromonas gingivalis*, *Prevotella intermedia*, and *Prevotella melaninogenica* in the serum and synovial fluid of RA patients, suggesting a potential link between periodontal infection and the development progress of RA.

*P. gingivalis*, one of the red complex bacteria, has an enzyme named *P. gingivalis Peptidylarginine Deiminase* (PPAD). This enzyme is capable of deiminating the C-terminal arginine residues of peptides and proteins in the post-translation modification process called citrullination, which can trigger the primary auto-immunization in the periodontal site known to be Anti-Citrullination Protein Antibodies (ACPA) [2,7,10,11]. In recent years, there has been a growing emphasis on the role of citrullination and autoantibodies in the development of RA. The production of ACPA plays a significant role in the pathogenesis of RA. ACPA levels serve as a highly specific marker for the disease, with specificity ranging from 95% to 98% [12]. These antibodies have been detected in the serum of approximately 70% of RA patients up to ten years before an accurate diagnosis is made [2]. Studies have also conducted to investigate relationship between anti-Pg and RA antibodies of Japanese. It was revealed that anti-Pg was associated with rheumatoid factor (RF), but not with anti-Cyclic Citrullinated Peptide (aCCP) [13]. In contrast to the ACPA, RF has only limited specificity and can be detected in several other diseases. It has been shown that RF by itself does not contribute to the progression of RA [2]. While *P. gingivalis* has been extensively studied as the primary microorganism associated with PD in the pathogenesis of RA, a recent investigation has identified another periodontal pathogenic microorganism, *Aggregatibacter actinomycetemcomitans*, as a potential trigger for RA development, establishing a novel link with PD. This study revealed that *A. actinomycetemcomitans* induced hypercitrullination in host neutrophils through dysregulated activation of citrullinating enzymes mediated by leukotoxin A (LtxA), a major virulence factor of *A. actinomycetemcomitans*. Consequently, this led to the formation of a citrullinome that mirrors the local citrullination observed in joints affected by RA [2,7]. Additionally, the study illustrated that LtxA prompted alterations in neutrophil morphology, resulting in the release of citrullinated proteins. Moreover, exposure to leukotoxic *A. actinomycetemcomitans* was confirmed in RA patients with PD and was found to be positively correlated with levels of ACPA [9]. It also has been shown that patients with RA exhibit a higher bacterial load, an increased presence of pathogenic species, and greater diversity in their oral microbiota compared to healthy controls, both with and without PD. This led to a deteriorated periodontal condition, as evidenced by clinical attachment loss in these RA patients. Furthermore, alterations in the oral microbiome, characterized by an increase in pathogenic species such as *Prevotella*, *A. actinomycetemcomitans*, and *Parvimonas micra*, were linked to poorer RA outcomes, including an increased number of tender and swollen joints, in patients with RA and PD [14].

In this regard, citrullination is known to be the link between RA and PD due to the expression of PPAD from *P. gingivalis* and the ability of LtxA in inducing citrullination process [15]. Numerous additional studies have suggested the potential implication of periodontitis-related bacteria, such as *P. intermedia* [16], *Tannerella forsythia* [16,17], *Cryptobacterium curtum* [18], *Leptotrichia* spp. [19], *Megasphaera* spp. [19,20], *Anaeroglobus geminatus* [19], *Veillonella* spp. [20,21], and *Granulicatella* spp. [20]. This study is conducted to investigate whether periodontitis-related bacteria other than *P. gingivalis* and *A. actinomycetemcomitans* might be associated with RA marked by disease parameters such as ACPA, aCCP, and RF. Furthermore, this review aims to assess the potential associations between non-periodontal bacterial species and clinical parameters of RA.

## 2. Materials and Methods

In this systematic review, we followed the principles outlined in the 2020 Preferred Reporting Items for Systematic Reviews and Meta-Analysis-PRISMA Statement [22]. The PICO question was formulated as follows: “Are there any associations between oral microbiota regarding periodontal disease and disease parameters of rheumatoid arthritis?” Population (P), in this review, includes all periodontitis patients with rheumatoid arthritis, with no Intervention (I). We also include healthy controls as Comparison (C) in order to define the association between the two diseases. As for the outcomes (O), we refer to oral microbiome abundance, periodontopathogen, disease status: periodontitis, rheumatoid arthritis; and RA disease parameters: ACPA/aCCP/RF.

The searches were limited to articles in English conducted in human participants. Cross-sectional and case–control studies analyzing oral microbiome and RA disease parameters using sequencing, polymerase chain reaction (PCR), or enzyme-linked immunosorbent assay (ELISA) methods were included. The exclusion criteria for this study were animal studies, case report studies, case series studies, and review articles. Studies presented in the form of abstracts at scientific conferences and other unpublished reports were excluded from the analysis.

### 2.1. Study Protocol and Registration

A study protocol was developed using the guidelines of the Preferred Reporting Items for Systematic Reviews and Meta-Analyses Protocols (PRISMA) 2020 [22]. The authors enrolled in the systematic review in the Prospective Register of Systematic Reviews (PROSPERO) at the University of York. The study was assigned the registration number CRD42024526527.

### 2.2. Search Strategy

The literature search was performed in electronic databases up to March 2024 using predetermined terms and strategies specific to each database (PUBMED, SCOPUS, and SCIENCE DIRECT) by two investigators (LA and IG) independently. The screening results were independently collected by two investigators and subsequently compared for consistency. To obtain specific results, we used the “advanced search” feature with keywords “rheumatoid arthritis OR joint disease AND oral microbiome OR porphyromonas gingivalis OR aggregatibacter actinomycetemcomitans OR treponema denticola OR tannerella forsythia OR anti-citrullinated protein antibody OR rheumatoid factor OR anti-cyclic citrullinated peptides” in the PUBMED database. Keywords for the SCOPUS database were “((rheumatoid AND arthritis) AND (periodontitis) OR (oral AND microbiome) OR (porphyromonas AND gingivalis) OR (tannerella AND forsythia) OR (treponema AND denticola) OR (anti-citrullinated AND protein AND antibody) OR (anti-citrullinated AND protein AND antibody) OR (anti-cyclic AND citrullinated AND peptides) OR (rheumatoid AND factor))”. To obtain appropriate papers from the SCIENCE DIRECT database, we used the keywords “(rheumatoid arthritis OR joint disease) AND (oral microbiome OR porphyromonas gingivalis OR aggregatibacter actinomycetemcomitans OR tannerella forsythia OR treponema denticola) AND anti-citrullinated protein antibody OR rheumatoid factor)”. Publication years were limited from 2014 to 2024. Furthermore, a supplementary online hand-search of recent publications from prominent periodontal microbiology and biological science journals over the past five years was conducted.

### 2.3. Risk of Bias

A risk of bias assessment was conducted to evaluate the quality of all selected reports using the Joanna Briggs Institute (JBI) critical appraisal tool for case–control and cross-sectional studies by two investigators (LA and IG). If there was any dispute regarding the risk of bias assesment, the third investigator (YS) was asked to review and determine the result. All checklists for the study are available at: https://jbi.global/critical-appraisal-tools (accessed on 27 September 2024). Information and explanation for all the study criteria are based on Moola et al. [23].

## 3. Results

### 3.1. Overview Search Process

**Figure 1:**
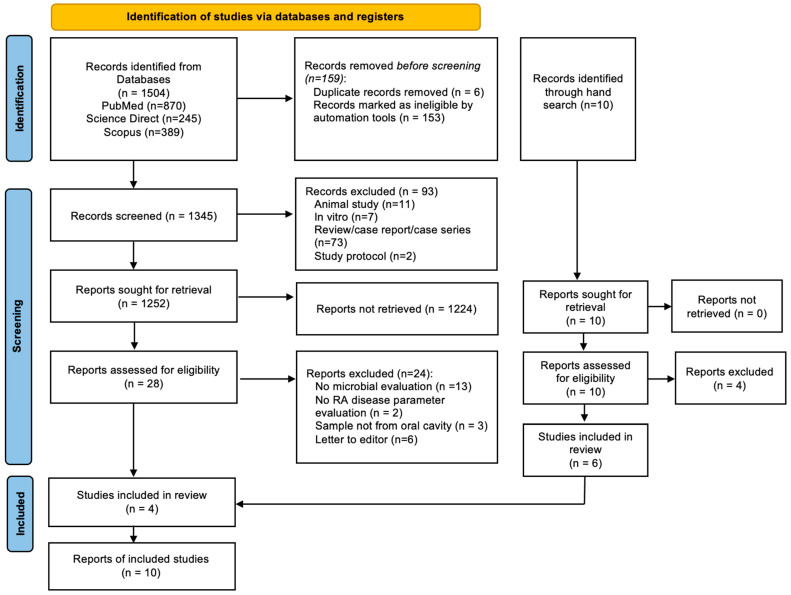
PRISMA 2020 flowchart for study selection.

During the initial article search conducted by the authors, a total of 1504 papers were identified and retrieved from selected databases, and 10 papers were identified by manual search. However, a total of 6 duplicate papers were identified, and 153 papers were not eligible due to incompatibility with keywords and thus had to be removed. This was then followed by a thorough screening using the inclusion and exclusion criteria, which led to the elimination of 93 papers. A total of 1252 remaining articles were sought for retrieval and 1224 more papers had to be excluded because they failed to meet the inclusion and exclusion criteria. An amount of 28 papers were assessed for eligibility and full-text evaluation and 24 more to be excluded. Following thorough deliberation, 10 articles were deemed eligible for inclusion in this systematic review, identified through both electronic database searches and manual screening. Figure 1 shows the PRISMA flowchart used for the study selection and the excluded report is provided in the Supplementary Table S1.

### 3.2. Studies Included

A total of ten studies were published between 2014 and 2023, including five case–control [21,24,25,26,27] and five cross-sectional designs [14,28,29,30,31]. Several studies included in this review examined the relationship between periodontal and non-periodontal bacteria and RA disease parameters, such as ACPA, aCCP-2, and RF. Participants included individuals with diagnosed RA with or without PD as well as healthy controls. All the studies included in this review fulfilled the predefined inclusion criteria.

### 3.3. Quality of the Studies

**Figure 2:**
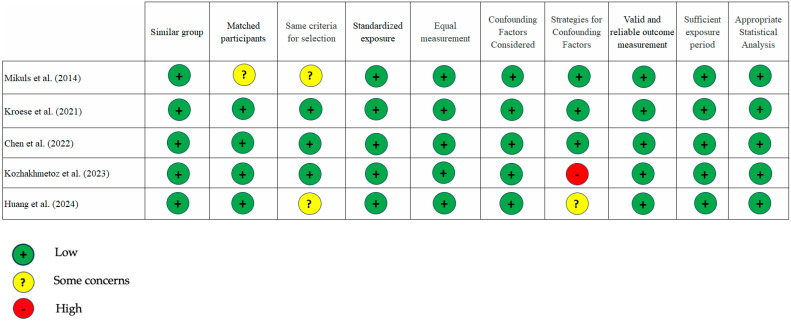
JBI Risk of bias plot for case–control studies, two studies showed “some concern” and one “high risk” risk of bias [21,24,25,26,27].

**Figure 3:**
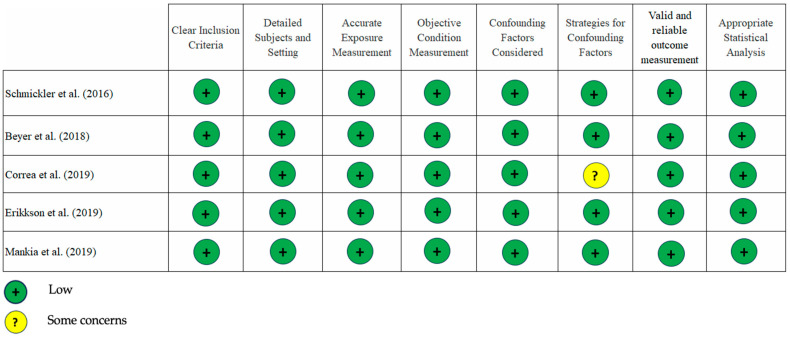
JBI risk of bias plot for cross-sectional studies showed only one study had “some concerns” risk of bias [14,28,29,30,31].

Our assessment using the JBI critical appraisal tool showed a low risk of bias for most studies included. There were very few instances of “some concern” and only one with a “high” risk of bias (Figure 2 and Figure 3). An area that needs attention is the evaluation of confounding factors, as the data analysis from the three studies [14,26,27] did not include sampling matching design or regression analysis. In our opinion, the overall quality of the studies included is good.

### 3.4. The Description of Study Characteristics

**Figure 4:**
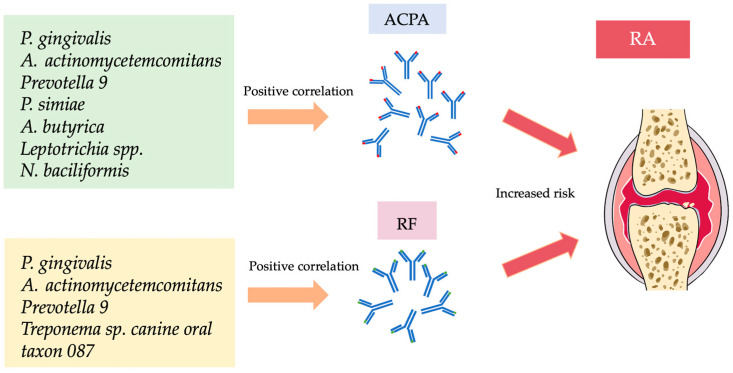
Positive correlation of periodontal pathogens with ACPA and RF that may increase the risk of RA. ACPA, anti-citrullinated protein antibodies; RF, rheumatoid factor; RA, rheumatoid arthritis.

The study characteristics and findings were described in Table 1. Three studies [24,26,27] identified correlations between periodontal bacteria and RA disease parameters, while two additional studies reported associations involving non-periodontal bacteria [25,26]. Mikuls et al. observed a weak correlation between *P. gingivalis* and both anti-CCP-2 and RF [24]. Similarly, Kozhakhmetov et al. found positive correlations between the periodontal bacteria *Prevotella_9* and *Treponema* sp. canine oral taxon 087 with ACPA and RF [26], while Huang et al. reported a positive association between *A. actinomycetemcomitans* and both aCCP and ACPA levels [27]. Mikuls et al. [24] observed ACPA overexpression in patients with subgingival *P. gingivalis* regardless of smoking status, while Eriksson et al. [30] reported a significantly higher prevalence of ACPA positivity in RA patients with moderate to severe PD compared to those with no or mild disease. Other studies focused on the dominant oral bacteria in RA patients with PD [14,21,28,29,30,31], rather than their relationship with RA parameters. In addition to identifying the dominant bacterial species in RA patients with PD, Correa et al. reported a significant association between *F. fastidiosum*, *P. micra*, and *A. geminatus* with an increased number of swollen joints [14]. Table 2 presents the conclusions of studies that identified a positive correlation between either periodontal or non-periodontal bacteria and RA disease parameters, specifically ACPA, aCCP-2, and RF [24,25,26,27,30]. Figure 4 shows positive associations of ACPA and RF with periodontal as well as non-periodontal bacteria.

## 4. Discussion

The identification of a protein known as peptidyl arginine deaminase, which possesses citrullination activity, in *P. gingivalis*, led to the study of the potential correlation between RA and PD. RA is believed to begin in inflammatory mucosal tissues, such as the lung and oral cavity (specifically the periodontal tissue), rather than within the joint itself. This, combined with genetic predisposition and environmental factors like smoking, contributes to the development of RA [32]. A study by de Smit et al. suggested that PD and specific bacterial taxas are linked to the existence of ACPA in the periodontium, which supports the idea that the development of RA may originate from mucosal tissues [33]. We conducted this study to further explore the evidence linking periodontitis-related bacteria to RA disease parameters like ACPA/aCCP and RF. Ten studies, consisting of five case–control studies and five cross-sectional studies, were included in this systematic review. Those ten studies investigated populations with RA, early RA, RA at risk with or without PD, and healthy controls. Samples were taken from subgingival biofilms and buccal epithelial cells and subsequently analyzed by real-time/quantitative polymerase chain reaction (RT/qPCR) or sequencing methods.

Real-time/quantitative PCR (RT/qPCR) is currently a widely accepted and recognized technique for identifying, measuring, and categorizing various microorganisms in the fields of clinical and veterinary diagnostics, as well as food safety. PCR, due to its ability to amplify a precise segment of DNA, has been employed in pathogen detection. qPCR is a powerful technique for analyzing DNA because it is fast, high-throughput, safer, accurate, and flexible [34,35]. PCR has limits despite being a useful method. PCR is an extremely sensitive technology; even a small amount of DNA contamination in the sample might lead to inaccurate results. Furthermore, to create primers for PCR, it is necessary to have preexisting sequence data. PCR is limited to detecting the existence or non-existence of a certain pathogen or gene. Another constraint is that the primers employed for PCR have the potential to bind to sequences that are similar, while not quite identical, to the target DNA, resulting in non-specific annealing. Furthermore, the DNA polymerase has the potential to introduce erroneous nucleotides into the PCR sequence, albeit with a minimal frequency [36].

Sequencing technology enables the detection of pathogens using qualitative or quantitative methods. Various sequencing techniques are employed to identify bacteria, such as 16SrRNA, whole-genome sequencing (WGS), and shotgun metagenomics (37,38). Next-Generation Sequencing (NGS) technology in clinical microbiology and infectious diseases provides a variety of advantages, including high throughput, diagnostic gold standard, enhanced detection, epidemiological tracking, and predictability of antimicrobial resistance. To put it in simple terms, NGS technology improves the comprehension of infectious diseases, epidemiological surveillance, and diagnostic capabilities [37].

Table 1 shows RT/qPCR has detected certain bacteria related to periodontitis, such as *P. gingivalis*, *F. nucleatum*, *P. intermedia*, and other periodontopathogens [20], and *A. actinomycetemcomitans* [32]. Higher CRP concentrations were reported in subjects with *P. gingivalis* in biofilms than in those without [21], and *A. actinomycetemcomitans* was positively correlated with aCCP, RF, ACPA, hs-CRP, and DAS28 [27]. As previously described by numerous studies, the association between *P. gingivalis* and RA is due to PPAD-induced citrullinated protein, which leads to ACPA production that can be detected in the serum or GCF of RA patients [2,7,10,11,15]. Interestingly, *P. intermedia*, *P. gingivalis*, *F. nucleatum*, and *Serratia proteamaculans* were found to be detected in synovial fluid patients with RA [38,39,40]. These findings suggest that periodontitis-related bacteria could have a role in RA etiology and explain that the secretion of PPAD by *P. gingivalis*, which resides in epithelial cells, can potentially have a citrullinating effect in remote areas of the periodontium or even in distant tissues [15]. Anti-*P. gingivalis* has a weak but statistically significant association with another RA disease parameter, RF, reported by Mikuls et al. [24]. Gingipains, an enzyme produced by *P. gingivalis*, can cleave IgG in the Fc region, converting it into RF antigens. This process may contribute to the development of RF antibodies in RA patients [11]. In addition, PAD-induced antigens lead to the production of RF-containing immune complexes [41].

More periodontitis-related bacteria were found in RA patients identified by sequencing techniques (Table 1), *including Fusobacterium*, *Prevotella*, *Corynebacterium*, *Actinomyces*, *Leptotrichia*, *Selemonas*, *Veillonella*, *Treponema*, *P. gingivalis* [21], *Actinomyces*, *Fretibacterium fastidiosum*, *Parvimonas micra*, *Anaeroglobus geminatus*, *Prevotella* spp., *A. actinomycetemcommitans* [14], *Prevotella oris, Porphyromonas* spp., *Desulfobulbus* spp., *Prevotella* spp., *Capnocytophaga* spp., *Tannerella* spp., *P. gingivalis* [30], *Prevotella salivae*, *Veillonella*, *Prevotella* spp. [21], *A. butyrica*, *P. simiae* [25], *Prevotella_9*, *Leptotrichia* spp., *N. baciliformis*, and *Treponema* sp. *canine oral taxon 087* [26]. The increase in certain bacteria in RA patients may be due to their disease status and therapy. Research by Beyer et al. [29] showed an increase in *Corynebacterium matruchotii, Actinomyces, Veillonella*, and *Streptococcus* bacteria in individuals who had active RA compared to RA disease in remission. These findings suggest that the subgingival microbiome composition is influenced by the disease activity status of RA patients, with active disease associated with specific microbial profiles. There is no detailed explanation of the relationship between the bacterial abundance in active RA and disease parameters, but we would like to highlight the indirect association between *Veillonella* and *P. gingivalis*. *Veillonella* produces menaquinone (vit K2), which is utilized by *P. gingivalis* and *Prevotella* [42]. In addition, *Veillonella* has a surface protein called hag1, which has a role in the coaggregation of bacteria in biofilms, including the late colonizer, *P. gingivalis* [43]. In our opinion, these abilities of *Veillonella* are thought to be one of many causes for *P. gingivalis*, a bacterium that plays a role in RA, to become prominent in biofilms and subsequently produce PPAD, which induces ACPA production.

Chen et al. [25] (Table 2) suggested that *A. butyrica* and *P. simiae* may have a significant role in the progression of RA. *A. butyrica* and *P. simiae* exhibited a significantly higher prevalence in patients with RA and showed a positive correlation with ACPA, indicating that they might serve as an initiator for ACPA development. The research also discovered that *A. butyrica* has the gene for making arginine deiminase, an enzyme that can make citrulline. On the other hand, *P. simiae* can increase the expression of endogenous PAD enzymes, which leads to proteins being citrullinated too much and the production of ACPA. These findings are intriguing because, according to the authors’ understanding, both *A. butyrica* and *P. simiae* are bacteria that are infrequently linked to periodontitis. *A. butyrica* is a unique anaerobic bacterium that has a significant function in the decomposition of amino acids and the generation of volatile fatty acids, which are also present in cattle waste [44]. *P. simiae* metabolizes a range of carbohydrates, such as glucose, fructose, and lactose, and generates acetate, butyrate, and propionate as its primary products of fermentation [45]. Cai et al. discovered the presence of *P. simiae* in the microenvironment of oral cavity squamous cell carcinoma (OSCC) [46].

*Leptotrichia* spp. and *Prevotella* were found to be abundant in new-onset RA patients by Scher et al. [47]. Meanwhile, Kozhakhmetov et al. (Table 2) described the association of oral microbiota, including *Prevotella_9*, *Leptotrichia* spp., *L. wadei*, *N. baciliformis*, and *Treponema* sp. canine oral taxon 087, with the disease parameters of RA [26]. The study indicates a correlation between *Prevotella_9* and elevated levels of ACPA and RF, suggesting a possible connection between these bacterial taxa and the synthesis of citrullinated proteins; nevertheless, its precise mechanism is not yet comprehensively understood. *Oscillospiraceae UCG-005*, *Leptotrichia* spp., *L. wadei*, and *N. baciliformis* moderately positively correlated with high ACPA levels, while *Treponema* sp. canine oral taxon 087 moderately positively correlated with high RF. Another interesting finding was that *A. geminatus* was significantly correlated with ACPA and RF levels [48], and moderately positively correlated with CRP [31]. More research into the ACPA+/RF− and ACPA+/RF+ subgroups found that those groups had a higher abundance of bacteria that metabolize glycosaminoglycans, which are crucial constituents of joint tissue [26]. These findings indicate that the oral microbiota may play a role in causing inflammation and damage to joint tissue in RA by transferring certain types of bacteria between the oral cavity and the gut, as well as causing greater permeability in the intestines during the later stages of RA [43]. To put it simply, all of these bacteria can trigger an inflammatory response and play a role in immune system dysregulation, leading to the formation of autoantibodies related to RA.

Huang et al. [27] (Table 2) showed that *A. actinomycetemcomitans* has a positive correlation with the RA disease parameters in RA and PD patients, including aCCP, RF, and ACPA. The explanation of association has been described previously as follows: Ltx-A, which is generated by *A. actinomycetemcomitans*, is the molecular mechanism that causes the deregulation and activation of citrullination in neutrophils. Ltx-A can imitate the process of membrane disruption, which helps to maintain the citrullination process of autoantigens in the joints affected by RA. This results in alterations in the structure of neutrophils and promotes the creation of extracellular traps, which then release cargo that is heavily citrullinated. It has been shown that increased levels of anti-Ltx-A antibodies are linked to the presence of ACPAs and RF in the plasma of individuals with RA [9,48,49]. Volkov et al. asserted that anti-LtxA levels likely provide a more precise depiction of subgingival *A. actinomycetemcomitans* infection [50]. Nevertheless, the existing research that establishes a connection between *A. actinomycetemcomitans* and RA is insufficient to definitively ascertain its pathogenic importance for the disease.

There have been many studies conducted on the relationship between RA and PD, particularly between RA and periopathogens. Recent systematic reviews and meta-analyses have shown an association between RA and *P. gingivalis*. A total of 28 papers reviewed based on studies conducted in Europe, North/South America, and Asia showed Odds Ratios (ORs) ranging from 0.33 to 14.6 (95% CI). The results also indicate that there are geographical variations in the association between exposure to *P. gingivalis* and RA. Specifically, the combined odds ratios from populations in Europe and North America were found to be significantly greater than those in Asia [51]. Although some studies have linked *P. gingivalis* to the level of RA autoantibodies, there are different findings. Scher et al. [47] found that the presence and abundance of *P. gingivalis* did not correlate with ACPA levels. Beyer et al. [29] also found no correlation between *P. gingivalis* with ACPA and RF in serum of RA patients. The potential correlation between subgingival *P. gingivalis* and ACPA also failed to be found by Rahajoe et al. [52]. This difference may be due to differences in analysis techniques, the source of samples taken, and the number of samples included in the study. Periodontal treatment and ongoing RA treatment may also have an influence on the results of the study, because both can provide changes to periodontal conditions and RA disease parameters [10].

The study had some limitations, one of which was that mostly subgingival biofilm samples were analyzed in this systematic review, whereas samples of GCF were not obtained. This decision was made based on the fact that periodontitis-related bacteria are more prevalent in the subgingival area. Furthermore, the ACPA/aCCP value analyzed in this study was exclusively derived from the blood serum of patients with RA, as serum ACPA value is a widely accepted diagnostic test with high specificity for determining RA cases [53]. We could not ignore the possibility that RA might arise from mucosal inflammation (periodontitis), which is characterized by elevated IgA ACPA in GCF [33,54]. We acknowledge that there may be heterogeneity in the data, which could impact our results. This may be attributed to factors such as population characteristics, sample size, and the varying classifications of periodontitis used across the studies. The findings of this review emphasize associations rather than causality.

Further research is needed in order to verify or strengthen the relationship between the two diseases. As this is resolved, we perform an early detection of RA by periodontal examination, thus allowing earlier diagnosis and intervention. If particular periodontal infections or inflammatory pathways are identified in this association, treatments aimed at those components could be investigated. Research that can be developed, for example, is finding specific proteins or virulence factors that play a role in the oral bacteria that are abundant in RA, so that the number of bacteria can be controlled through such manipulation, then treatment of RA by performing microbiome-specific therapy becomes possible in the future. This understanding could be advantageous in the development of more efficacious medications for RA, even if they do not specifically target the oral cavity.

## 5. Conclusions

In conclusion, *P. gingivalis* and *A. actinomycetemcomitans* are associated with elevated levels of serum ACPA/aCCP and RF in RA with periodontitis patients. Furthermore, there were several bacteria in RA patients that showed a possible correlation with serum ACPA, including *A. butyrica*, *P. simiae*, *Oscillospiraceae* UCG-005, *Leptotrichia* spp., *L. wadei*, and *N. baciliformis*. On the other hand, *Treponema* sp. *canine oral taxon 087* may be to be associated with RF levels. There was also a positive association between *Prevotella_9* and the levels of both ACPA and RF in the serum of patients with RA. Future comprehensive examinations of the oral microbiome and the molecular pathways employed by oral bacteria are expected to facilitate the development of innovative therapies and diagnostic tools for PD and RA.

## Figures and Tables

**Table 1:** Study characteristics and findings.

No.	Author (Country) Study Type	Population/ Condition	Microbial Sampling Source/Assay	Rheumatoid Parameter/Disease Activity	Main Findings
1.	Mikuls et al. [24] USA 2014 Case–control	1. RA (n = 287); age 59 ± 12; m/f 63%/37%, BMI: 30 ± 7 kg/m^2^ 2. OA (n = 330) age 59 ± 11; m/f 60%/40%, BMI: 32 ± 7 kg/m^2^	Biofilm subgingiva PCR	Anti-CCP-2 ≥ 5 units/mL RA-PD vs. RA non PD = 90% (170 ± 128 unit/mL) vs. 82% (131 ± 126 unit/mL) RF > 15 IU/mL RA-PD vs. RA non PD = 86% (390 ± 633 IU/mL) vs. 72% (185 ± 379 IU/mL)	1. PD patients have a higher chance of being RF positive (*p* = 0.006) and concentrations of RF and anti-CCP-2 (*p* < 0.05) compared to those without PD. 2. The levels of antibody against *F. nucleatum* significantly higher in RA cases (*p* = 0.018). 3. The anti–*P. gingivalis* statistically significant and weakly correlate with anti–CCP-2 (r = 0.14, *p* = 0.022) and RF (r = 0.19, *p* = 0.001) 4. Over-expression of ACPAs of patients with subgingival *P. gingivalis*, regardless of smoking status.
2.	Schmickler, et al. [28] Germany 2016 Cross-sectional	1. RA (n = 168); age 58.4 ± 7.9; m/f: 18.5%/81.5% 2. Healthy patients (n = 168); age 56.8 ± 6.8; m/f: 39.9%/60.1%	Biofilm subgingiva RT-PCR	RF positive Positive: ≥20 U/mL Negative: <20 U/mL RF positivity: 35% aCCP Positive: ≥5 U/mL Negative: <5 U/mL aCCP positivity: 44%	1. *F. nucleatum* (*p* < 0.001) and *P. micra* (*p* < 0.001) more prevalent in RA group. 2. There were no significant differences in the concentration of *P. gingivalis* (*p* = 0.06) and *F. nucleatum* (*p* = 0.06) in aCCP-positive patients and *P. intermedia* (*p* = 0.08) in RF-positive patients.
3.	Beyer et al. [29] Norway 2018 Cross-sectional	Chronic RA (n = 78), aged 57 ± 11.5; were divided into 4 subgroups: 1. Gingivitis (n = 14) 2. Mild periodontitis (n = 7) 3. Moderate periodontitis (n = 43) 4. Severe periodontitis (n = 14)	Biofilm subgingiva Sequencing qPCR	ACPA positive: >3 U/mL RF posisitve: >25 IU/mL	1. The most predominant genera: *Fusobacterium, Prevotella*, *Corynebacterium*, *Actinomyces*, *Leptotrichia*, *Selenomonas*, *Veillonella*, and *Treponema*. 2. Higher CRP in subjects with subgingival *P. gingivalis* (*p* = 0.009). 3. Microbial related to higher RA disease duration, disease activity, prednisolone dose, and ESR: *Corynebacterium*, *Veillonella*, *Actinomyces*, *Leptotrichia*, *Streptococcus* and *Neisseria*.
4.	Correa et al. [14] Brazil 2019 Cross-sectional	1. RA with PD (n = 21); m/f: 66%/34%; 53 ± 10.4 2. RA non PD (n = 21); m/f: 46.52%/53.48%; 50 ± 11.1 3. Non CP (control) (n = 27); m/f: 63.5%/36.5%; 42.8 ± 14 4. CP (control) (n = 20); m/f: 52%/48%; 46.5 ± 12.3	Biofilm subgingiva Next Generation Sequencing (NGS)	ACPA positive: ≥5 U/mL RA non-CP vs. RA + CP: 33% vs. 85.7% DAS28 (tender and swollen joints) RA non-CP vs. RA + CP: 3.5 ± 1.2 vs. 3.7 ± 1.5	1. *Fretibacterium fastidiosum, Parvimonas micra* and *Anaeroglobus geminatus* were correlated with augmented numbers of swollen (rho = 0.35) and tender joints (rho = 0.30, *p* < 0.05). 2. Elevated numbers of *Prevotella* spp., *A. actinomycetemcommitans*, and *P.micra* in RA + PD.
5.	Eriksson et al. [30] Sweden 2019 Cross-sectional	1. RA with no/mild PD (n = 10); aged: 50 + 14; m/f: 0%/100%; BMI26 ± 6.8 2. RA with moderate/severe PD (n = 30); aged: 64 ± 7.8; m/f: 17%/83%; BMI: 24 ± 5.6	Biofilm subgingiva Sequencing qPCR	ACPA positive: 3 E/mL RF positive: 20 E/mL No/mild periodontitis ACPA-positive (50%) RF-positive (50%) Moderate/severe periodontitis ACPA-positive (86%) RF-positive (73%)	1. *Prevotella oris* and *Porphyromonas* sp. are abundant in mild/non PD group. 2. Moderate/severe PD includes numerous bacteria: *Desulfobulbus* sp. *prevotella, NA 92, Bulleidia, Capnocytophaga*, and *Tannerrella.* 3. There were no significant differences in the percentage of *P. gingivalis* (*p* = 0.66) between the moderate/severe PD group and the no/mild PD group. 4. The prevalence of ACPA positive was substantially higher (*p* = 0.032) in RA patients with moderate/severe PD compared to those with no/mild PD.
6.	Mankia et al. [31] United Kingdom 2019 Cross-sectional	1. Healthy control (n = 32); aged: 49.4 ± 15.3; m/f: 46%/54% 2. aCCP + at-risk (n = 48); aged: 51.9 ± 11.4; m/f: 35%/65% 3. Early RA (n = 26); aged: 54.4 + 16.7; m/f: 46%/54% Divided into 4 subgroups according to clinical periodontal status: 1. PD (n = 12) 2. Healthy (n = 10) 3. RA with PD (n = 14) 4. RA without PD (n = 3)	Biofilm subgingiva Shotgun metagenomic analysis	IgG anti CCP+ Positive: ≥3 times upper limit of normal range (BioRad)	There is a higher occurrence of PD and *P. gingivalis* in CCP+ at risk individuals.
7.	Kroese et al. [21] Netherland 2021 Case–control	1. Patients with early RA (n = 50); aged: 52.1 + 13.2; m/f: 22%/78% 2. Individual at risk of developing RA (n = 50); aged: 51.4 + 10.3; m/f: 24%/76%. 3. Healthy control (n = 50) aged: 51.2 + 11.0; m/f: 24%/76%.	Subgingival, saliva, tongue coating Sequencing	ACPA Positive: >10.0 kU/liter RF Positive: >5.0 kU/ liter ACPA positive Early RA/at risk of RA/healthy control: 62%/48%/0% RF positive Early RA/at risk of RA/healthy control: 74%/92%/0% Both ACPA and RF positive Early RA/at risk of RA/healthy control: 76%/100%/0%	1. There were no microbial significant differences in subgingival plaque, saliva, and tongue coating between ACPA (+) and (−) individual at risk of RA. 2. *Prevotella salivae, Veillonella (p* = 0.012) and *Prevotella* (*p* = 0.022) were more abundant in the early RA group and at-risk individual. 3. *Neisseria flavescens/subflava* (*p* = 0.02), *Porphyromonas pasteri*/sp._oral_taxon_278 (*p* = 0.01), and *Veillonella parvula* were more abundant in the healthy control group.
8.	Chen et al. [25] Taiwan 2022 Case–control	1. AM (age, gender, DM statuses) (n = 42; 21 RA, 21 control); aged 57.57 + 8.06, 67.19 + 8.73; m/f: 23.81%/76.19%. 2. PD (n = 24; 12 RA, 12 control); aged 59.00 + 7.8, 58.5 + 7.86; m/f: 25%/75%. 3. PH (n = 12; 6 RA, 6 control); aged: 54.50 + 11.66, 53.33 + 11.04; m/f: 16.67%/83.33%.	Biofilm subgingiva Sequencing	ACPA (Elia Kit CCP) ACPA level: AM (RA/control) vs. PD (RA/control) vs. PH (RA/control): 54.50/1.00 vs. 97.50/0.80 vs. 142.5/1.20 Positive: >10U/mL AM vs. PD vs. PH: 71.43% vs. 83.33% vs. 0 RF (N Latex RF Kit) Positive:>8 IU/mL AM (RA/control) vs. PD (RA/control) vs. PH (RA/control): 80.95%/9.52% vs. 91.67%/8.33% vs. 83.33%/16.67%	1. *A. butyrica* and *P. simiae* positively correlated with the levels of ACPA in AM + PD patients. 2. *A. butyrica* and *P. simiae*, have a potential role in the development of RA through the induction of ACPA production by hypercitrullination.
9.	Kozhakhmetov et al. [26] Kazakhtan 2023 Case–control	1. RA (n = 75); aged: 46 (mean); BMI: 24.5 (mean) 2. Control (n = 114); aged 43 (mean); BMI: 25.5 (mean)	Surface tongue, gums, tonsils, plaque Sequencing	ACPA positive: 57.3% RF positive: 76.0%	*1. Prevotella_9* positively correlated with ACPA and RF (*p* < 0.001) 2. *Oscillospiraceae* UCG-005, *Leptotrichia* spp., *Leptotrichia wadei*, *Neisseria baciliformis* positively correlated with of ACPA (*p* < 0.05) 3. *Treponema* sp. *canine oral taxon 087* positively correlated RF (*p* < 0.05)
10.	Huang et al. [27] 2023 Case–control	1. RA (n = 67); aged: 50.5% + 8.17; m/f:25.4%/74.6%; BMI: 23.1 + 2.83 2. RA + PD (n = 48); aged 49.9 + 8.74; m/f: 22.9%/77.1%; BMI: 24.2 + 3.63	Saliva and buccal epithelial cells (BECs) PCR	Anti-CCP level (U/mL): RA vs. RA + PD: 130.2 + 112.5 vs. 182.1 + 107.8 Anti-CCP positivity: RA vs. RA + PD: 59.7% vs. 81.3% RF concentration level (IU/mL): RA vs. RA + PD: 273.2 + 102.6 vs. 365.5 + 142.6 RF positivity: RA vs. RA + PD: 68.7% vs. 83.3% ACPA concentration level (IU/mL): RA vs. RA + PD: 498.2 + 598.7 vs. 778.1 + 842.0 ACPA positivity: RA vs. RA + PD: 56.7% vs. 75%	The *A actinomycetemcomitans* were positively correlated with anti-CCP (*p* = 0.008), RF concentration (*p* < 0.001), ACPA (*p* = 0.006).

RA, rheumatoid arthritis; m/f, male/female; BMI, body mass index; OA, osteoarthritis; PCR, polymerase chain reaction; anti-CCP-2, anti-cyclic citrullinated peptide-2; PD, periodontitis; AM, All Matched; DM, Diabetes Mellitus; RF, rheumatoid factor; IU/mL, international unit/mL; aCCP, anti- cyclic citrullinated peptide; U/mL, unit/mL; ACPA, anti-citrullinated protein antibodies; CP, chronic periodontitis; PH, periodontally healthy.

**Table 2:** Positive correlation of oral microbiota with disease parameters in RA.

Bacteria	ACPA	Anti-CCP2	RF	Author
*Porphyromonas gingivalis*		√	√	Mikuls et al. (2014) [24]
*Porphyromonas gingivalis*	√			Eriksson et al. (2019) [30]
*Aminipila butyrica*	√			Chen et al. (2022) [25]
*Peptococcus simiae*	√			Chen et al. (2022) [25]
*Prevotella_9*	√		√	Kozhakhmetov et al. (2023) [26]
*Leptotrichia* spp.	√			Kozhakhmetov et al. (2023) [26]
*Oscillospiraceae* UCG-005	√			Kozhakhmetov et al. (2023) [26]
*Leptotrichia wadei*	√			Kozhakhmetov et al. (2023) [26]
*Neisseria baciliformis*	√			Kozhakhmetov et al. (2023) [26]
*Treponema* sp. *canine oral taxon 087*			√	Kozhakhmetov et al. (2023) [26]
*Aggregatibacter actinomycetemcomitans*	√	√	√	Huang et al. (2023) [27]

ACPA, anti-citrullinated protein antibodies; anti-CCP-2, anti-cyclic citrullinated peptide-2; RF, rheumatoid factor.

## Data Availability

Data are available from the authors upon reasonable request.

## Supplementary Materials

The following supporting information can be downloaded at: https://www.mdpi.com/article/10.3390/dj13050214/s1, Table S1: Excluded Paper.

## Abbreviations

The following abbreviations are used in this manuscript

RA	Rheumatoid Arthritis
PD	Periodontal Disease
ACPA	Anti-Citrullinated Protein Antibodies
Anti-CCP2	Anti-Cyclic Citrullinated Peptide
RF	Rheumatoid Factor
IgA	Imunoglobulin A
PAD	Peptidyl-Arginine Deiminase
PPAD	*P. gingivalis* Peptidyl-Arginine Deiminase
Ltx-A	Leukotoxin A
GCF	Gingival Crevicular Fluid
RT/qPCR	Real-Time/quantitative polymerase chain reaction
hs-CRP	High sensitivity-C-Reactive Protein
DAS-28	Disease Activity Score-28
NGS	Next Generation Sequencing
WGS	Whole Genome Sequencing
DNA	Deoxyribonucleic Acid
BMI	Body Mass Index
